# Genome-Wide Discovery and Deployment of Insertions and Deletions Markers Provided Greater Insights on Species, Genomes, and Sections Relationships in the Genus *Arachis*

**DOI:** 10.3389/fpls.2017.02064

**Published:** 2017-12-19

**Authors:** Manish K. Vishwakarma, Sandip M. Kale, Manda Sriswathi, Talari Naresh, Yaduru Shasidhar, Vanika Garg, Manish K. Pandey, Rajeev K. Varshney

**Affiliations:** ^1^International Crops Research Institute for the Semi-Arid Tropics, Hyderabad, India; ^2^The University of Western Australia, Crawley, WA, Australia

**Keywords:** *Arachis*, InDel, genomes, phylogeny, sections

## Abstract

Small insertions and deletions (InDels) are the second most prevalent and the most abundant structural variations in plant genomes. In order to deploy these genetic variations for genetic analysis in genus *Arachis*, we conducted comparative analysis of the draft genome assemblies of both the diploid progenitor species of cultivated tetraploid groundnut (*Arachis hypogaea* L.) i.e., *Arachis duranensis* (A subgenome) and *Arachis ipaënsis* (B subgenome) and identified 515,223 InDels. These InDels include 269,973 insertions identified in *A. ipaënsis* against *A. duranensis* while 245,250 deletions in *A. duranensis* against *A. ipaënsis*. The majority of the InDels were of single bp (43.7%) and 2–10 bp (39.9%) while the remaining were >10 bp (16.4%). Phylogenetic analysis using genotyping data for 86 (40.19%) polymorphic markers grouped 96 diverse *Arachis* accessions into eight clusters mostly by the affinity of their genome. This study also provided evidence for the existence of “K” genome, although distinct from both the “A” and “B” genomes, but more similar to “B” genome. The complete homology between *A. monticola* and *A. hypogaea* tetraploid taxa showed a very similar genome composition. The above analysis has provided greater insights into the phylogenetic relationship among accessions, genomes, sub species and sections. These InDel markers are very useful resource for groundnut research community for genetic analysis and breeding applications.

## Introduction

The systematic development of different types of molecular markers over three decades through various approaches, such as storage proteins, isozymes, random amplified polymorphic DNA (RAPDs), restriction fragment length polymorphism (RFLP), amplified fragment length polymorphism (AFLP), cleaved amplified polymorphic sequence (CAPS), simple sequence repeat (SSR), start codon targeted polymorphism (SCoT), etc. helped in conducting genetic studies in several crops species including groundnut (Bechara et al., 2010; Pandey et al., 2012b; Varshney et al., 2013; Liu et al., 2015). Among different marker types, SSRs have dominated genetic studies in groundnut due to multiple advantages of SSR markers but lacked amenability for high throughput genotyping (Gupta and Varshney, 2000). Therefore, the current emphasis is now on developing single-nucleotide polymorphism (SNP) markers in groundnut due to their high preponderance throughout the genome and their amenability for high throughput genotyping for genome-wide breeding applications (Varshney et al., 2009; Pandey et al., 2016). Surprisingly, both SSRs and SNPs have their own drawbacks in spite of usefulness. For instance, the complicated and heterogenous mutation forms of SSRs can sometimes mislead the data analysis (Ellegren, 2004). Similarly, genotyping error may happen due to false bands and proficient artifacts (null or false alleles, allelic dropouts, size homoplasy) (Pompanon et al., 2005). Furthermore, regardless of many refined genotyping methods available (Syvänen, 2001), comparatively most of them are costly and needs special instruments for high throughput genotyping.

Insertions-deletions (InDels) are the second most prevalent and frequent structural variations detected across the plant genomes (Yang et al., 2014; Lu et al., 2015). InDels originate as a consequence of some cellular events like replication slippage, transposable elements, and crossing-over (Moghaddam et al., 2014). InDel process maintains beneficial as well as the deleterious effect on specific loci in the genome (Pearson et al., 2005). InDels are discernible as an efficient marker system for genetic studies in crops and a good addition to other sequence-based genetic markers mainly due to countless desirable inherent genetic ascribes as co-dominant inheritance and multi-allelic with genome-wide dispersion. In addition to easy detection in the genome, InDel markers can be selected by their anticipated fragment size and validated in genetic populations/germplasm using simple and cost-efficient agarose gel. These features make InDels an appropriate marker system for various translational genomics studies in crop plants.

Groundnut (*Arachis hypogaea* L.) is an economically important, nutritious and protein-rich oilseed crop grown in tropical and warm temperate regions of the world. The genus *Arachis* is indigenous to South America and its different species are widespread in >100 countries of the world and includes 80 species (Krapovickas and Gregory, 1994; Valls and Simpson, 2005). Based on the morphological variation, geographic distribution and ability to cross (cross compatibility), these species are grouped into nine sections namely *Arachis, Caulorrhizae, Erectoides, Extranervosae, Heteranthae, Procumbentes, Rhizomatosae, Triseminatae, and Trierectoides*. Of these sections, *Arachis* is the most diverse section with 30 different species including *A. hypogaea*, the cultivated groundnut species. Arachis section has much diversity at ploidy level i.e., two tetraploids (*A. hypogaea* and *A. monticola* with 2*n* = 4*x* = 40), three aneuploids (2*n* = 2*x* = 18; *A. decora, A. palustris, and A. praecox*) and remaining diploids (2*n* = 2*x* = 20; Valls and Simpson, 2005). The cultivated tetraploid, *A. hypogaea*, is considered to have originated from two diploid species namely *Arachis duranensis* (AA) and *Arachis ipaënsis* (BB) (Seijo et al., 2004). All the cultivated genotypes can be further grouped into subspecies (*hypogaea* and *fastigiata*), varieties (*aequatoriana, fastigiata, hirsuta, hypogaea, peruviana, and vulgaris)* and agronomic types (Spanish, Southeast-runner, Valencia, and Virginia) (Krapovickas and Gregory, 1994, 2007).

Albeit, with the boastful variation in phenotype, the species display less genetic diversity, as earlier reported with the analyses using RFLPs, SSRs, and SNPs (Pandey et al., 2012b; Varshney et al., 2013). Availability of genome sequences for both the diploid ancestors of tetraploid groundnut (Bertioli et al., 2016; Chen et al., 2016) fostered the process of genome resequencing of diverse cultivars. Therefore, this study reports *in silico* discovery of large-scale informative genome-wide InDels, development of InDel markers, validation and their deployment for conducting phylogenetic analysis of *Arachis* genus to understand the genetic complexity at the genome and species level.

## Materials and methods

### Plant materials and DNA isolation

A panel of 96 groundnut genotypes comprising of cultivated groundnut (*A. hypogaea* L.) and its wild relatives were used to evaluate InDel polymorphisms and their discriminatory power. The panel of 96 accessions had representation from 41 different *Arachis* species of seven sections and two synthetics viz. ISATGR278-18 (*A. duranensis* ICG 8138 × *A. batizocoi* ICG 13160) and ISATGR184 (*A. duranensis* ICG 8128 × *A. ipaënsis* ICG 8206) (Supplementary Table 1). Genomic DNA isolation using the modified CTAB method (Doyle and Doyle, 1987) from freshly collected unopened leaves followed by DNA quantification was performed following the protocol explained in Mace et al. (2003).

### Accessing genome sequences and genome-wide indels discovery

Considering the similarities between two diploid subgenomes of tetraploid groundnut (Bertioli et al., 2016), the genome sequences of *A. duranensis* (accession V14167 for A subgenome,) and *A. ipaënsis* (accession K30076 for B subgenome,) were used for mining the InDels using the methodology depicted by Yang et al. (2014). Briefly, the fasta files for A and B subgenomes were downloaded from PeanutBase site (https://www.peanutbase.org/) and InDels were identified using MUGSY software (Angiuoli and Salzberg, 2011). The alignment results were mined for InDel polymorphism using the Perl scripts provided by Dr. Wencai Yang (China Agricultural University, Beijing, China). Only the reads mapped on respective pseudomolecules of A and B subgenomes were considered for InDel discovery. The 100 bp (base pair) sequences flanking the candidate InDels were considered from A genome for insertion and from B genome for deletion. The low similarity sequences were removed by searching the extracted sequences against respective genomes using BLASTN program (Altschul et al., 1990) with an *E*-value of e-20. In house Perl script was used to extract (insertion or deletion) types, chromosomal position, and length of InDels (Supplementary Table 2). The Primers with desired features such as 100–200 bp product size and 20 bp optimum primer length were designed from the extracted sequences by using Primer3 software (Untergasser et al., 2012). The circular plots were constructed using standalone version of circos software (http://circos.ca/) developed at the Genome Sciences Centre in Vancouver, Canada (Krzywinski et al., 2009). The insertion and deletion densities are plotted at a window size of 100 Kb.

### InDel polymorphism and genotyping

Primer pairs were first used on four accessions/genotypes namely ICG 8138 (*A. duranensis*), ICG 8206 (*A. ipaënsis*), TAG 24 (*A. hypogaea*), and GPBD 4 (*A. hypogaea*). Subsequently, polymorphic InDels were used for the genotyping set of 96 diverse accessions. Each PCR reaction mixture of total volume 5 μl, contained ~5 ng of template DNA with 2 picomoles of each primer (forward and reverse), 2 mM of each dNTP, 2 mM MgCl_2_, 1X buffer (Kapa Biosystems) and 0.1 U of Taq DNA polymerase (Kapa Biosystems). PCR (ABI 9700 thermal cycler, Applied Biosystems, USA) reaction was performed following touchdown PCR program explained in Pandey et al. (2012a) and Varshney et al. (2014). The amplified PCR products of all accessions were electrophoresed on 1.5% agarose gels for 2 h at 80–100 V and visualized under gel documentation unit (Syngene). The amplified products were scored on the basis of presence (1) or absence (0) of alleles and the fragment sizes were determined by comparing the 100 bp DNA ladder band.

### Population structure and phylogenetic analysis

The population structure of the 96 *Arachis* accessions was analyzed utilizing genotyping data for polymorphic InDel markers using the program STRUCTURE 2.2 (Pritchard et al., 2000). The presumed number of k (the number of groups) was set from 1 to 10 with admixture and related frequency models; five independent simulations were executed for each range of values. Admixture and concerned frequency models including standard parameters such as 10,000 iterations before a burn-in length of 10,000 MCMC (Markov Chain Monte Carlo) replications for each simulation were used for population structure analysis. The optimal *k-*value was estimated by posterior probability [LnP(D)] and accessions were assigned to a representing group, as elucidated by Remington et al. (2001). Shannon–Weaver diversity index was analyzed using the GenAlEx 6.5 (Peakall and Smouse, 2012). An UPGMA dendrogram of the 96 accessions, sections and species of *Arachis* was constructed using DARwin 6 (Perrier et al., 2003). Dissimilarity matrix was generated from the binary data using Jaccard's coefficient at 1,000 bootstraps and the dendrogram was generated using UPGMA method. Dendroscope (Huson et al., 2007) was used to depict the relationship among them. Based on Nei's (1972) genetic distance, PIC-value was also calculated using PowerMarker 3.25 (Liu and Muse, 2005).

## Results

### Discovery of genome-wide InDels in two diploid ancestor genomes

**Figure 1:**
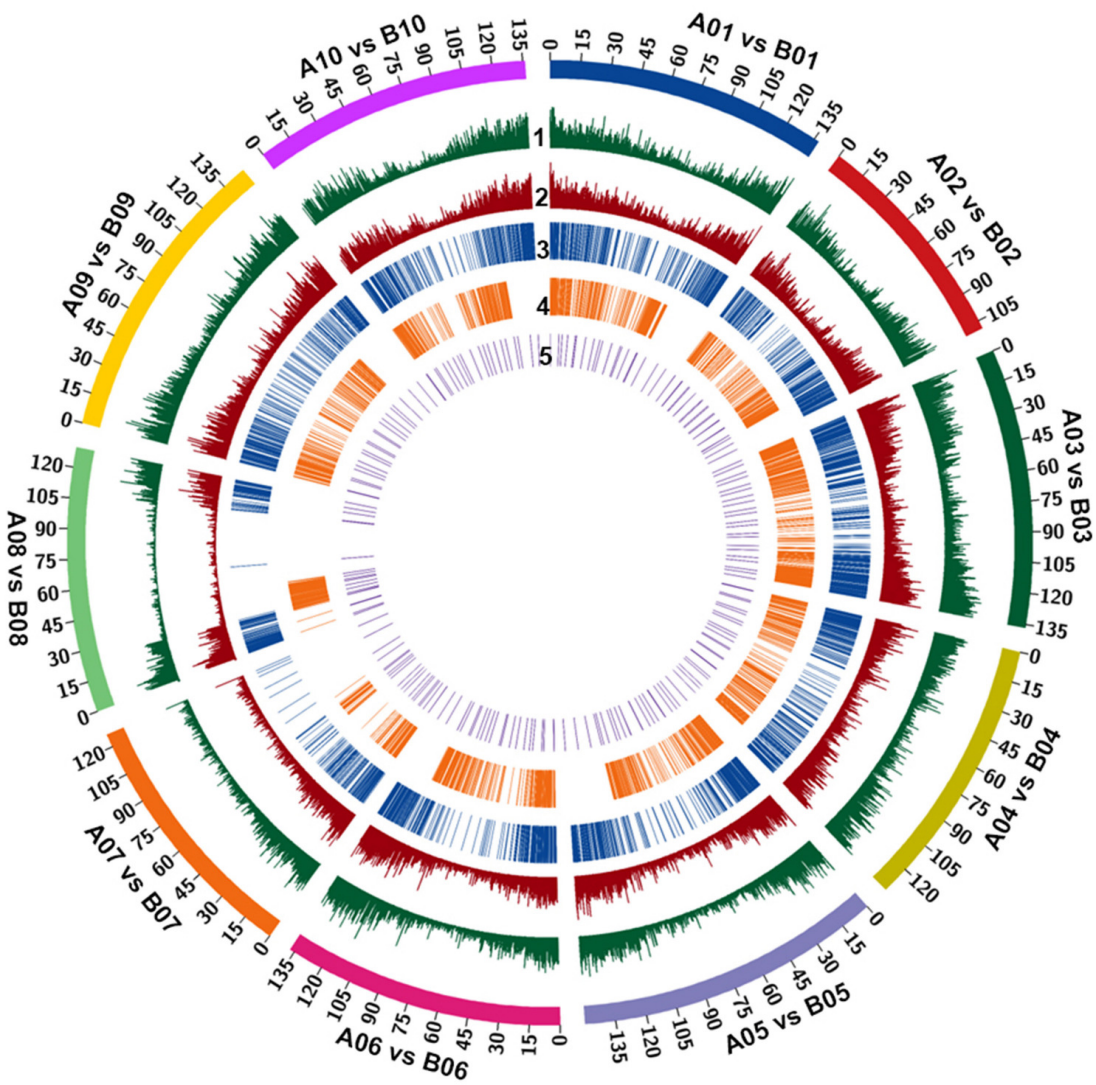
The frequency and relative distribution of 515,223 InDels physically mapped on A and B sub-genomes. The outermost circle denotes the different physical size (Mb) of 10 chromosomes of both sub-genomes coded with multiple colors as per the pseudomolecule size documented in groundnut genome. (1) All insertions density. (2) All deletions density. (3) insertions > 50. (4) deletions > 50. (5) Selected markers for genotyping.

**Figure 2:**
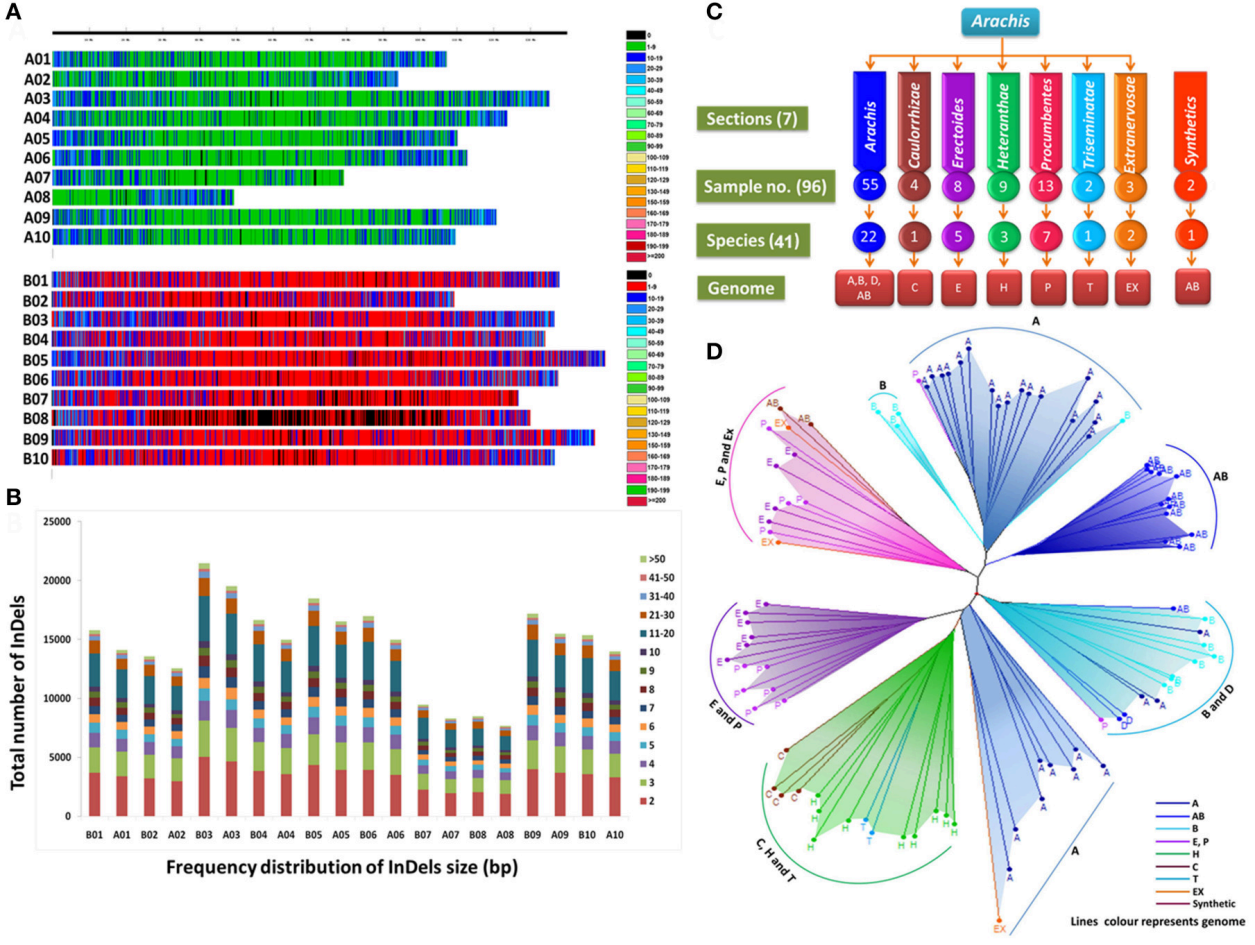
**(A)** InDels distribution pattern on A- and B- sub genomes. **(B)** Frequency distribution of InDels (bp) in *A. duranensis* and *A. ipaënsis*. **(C)** Number of samples taken for study from *Arachis* sections representatives of different species and genomes and **(D)** Grouping pattern of the accessions/genotypes based on polymorphic InDels.

A total of 269,973 insertions and 245,250 deletions were identified by comparing the genome assemblies of both the progenitor species i.e., *A. duranensis* (A subgenome) and *A. ipaënsis* (B subgenome). The lowest number of insertions (14,054) were detected between homeologous pseudomolecules A08 and B08, while the highest number of insertions (36,732) were detected between homeologous pseudomolecules A03 and B03. Similarly, the lowest number of deletions (12,849) were detected between homeologous pseudomolecules A08 and B08 while the highest number of deletions (33,537) were detected between homeologous pseudomolecules A03 and B03 (Table 1, Figures 1, 2A,B). The number of deletions were lower than the number of insertions across corresponding pseudomolecules (Figures 1, 2A,B). The average size of insertion was 4.69 bp with the range of 1–105 bp, while, the average size of deletion was 4.50 bp and varied from 1 to 105 bp. It was important to observe that the number of InDels decreased gradually with increase in size of InDels. For instance, ~43.7% InDels were of 1 bp size and 39.9% were of 2–10 bp while remaining 16.4% were of 10–50 bp.

**Table 1:** Summary statistics of InDels discovery, primer design, and polymorphisms.

**S. No**.	**Chromosome**	**Total InDels**	**Size (Mb)**	**>50 bp**	**Number of primers designed**	**No. of polymorphic primers**
**DELETIONS IDENTIFIED IN** ***A. duranensis*** **AGAINST** ***A. ipaënsis***						
1	A01	25,637	107.04	264	11	4
2	A02	21,850	93.87	219	11	6
3	A03	33,537	135.06	381	10	4
4	A04	26,770	123.56	266	11	8
5	A05	29,065	110.04	290	12	7
6	A06	26,758	112.75	281	11	1
7	A07	15,537	79.13	144	9	3
8	A08	12,849	49.46	139	9	5
9	A09	27,935	120.67	260	11	4
10	A10	25,312	109.46	236	10	3
**INSERTIONS IDENTIFIED IN** ***A. ipaënsis*** **AGAINST** ***A. duranensis***						
11	B01	28,240	137.41	305	10	4
12	B02	23,775	108.99	279	12	6
13	B03	36,732	136.11	489	11	5
14	B04	29,370	133.61	353	11	6
15	B05	32,040	149.90	390	11	5
16	B06	29,801	137.15	368	11	3
17	B07	17,354	126.35	167	11	1
18	B08	14,054	129.61	165	10	0
19	B09	30,768	147.09	356	11	6
20	B10	27,839	136.18	346	11	5
Total		515,223	2,383.44	5,698	214	86

### InDel marker development and their validation

In order to develop user-friendly markers, InDels >50 bp were selected for primer designing and marker development. A total of such 5,698 InDels (3,218 insertions and 2,480 deletions) with >50 bp size were selected for further analysis and primer designing. Out of 5,698 selected InDels, primers could be designed for 5,519 InDels i.e., 3,111 insertions and 2,408 deletions (Supplementary Tables 3, 4). A total of 214 evenly distributed InDel markers with >50 bp size were selected for amplification and polymorphism study in a set of diverse germplasm. For these selected InDels, the physical distance between two InDel markers ranged from 0.06 to 42.18 Mb with an average of 6.54 Mb. Out of these 214 InDel markers, 86 (40.19%) markers found polymorphic in these tested genotypes (Table 1). These polymorphic InDels with the precise amplification, clear amplicons and length polymorphism were further used to study the phylogeny of 96 diverse accessions. The polymorphic information content (PIC) values of InDel markers ranged from 0.006 (Ai.B01_137090268) to 0.9951 (Ad.A01_10274031) with an average of 0.490.

### InDel markers based genetic diversity among seven sections of *Arachis* genus

The 96 *Arachis* accessions, belonging to 41 species from seven sections and two synthetics, were assessed for allelic diversity using genotyping data of 86 polymorphic InDel markers (Table 1; Figure 2C). In total, 174 alleles were identified (2.02 alleles per marker). Among the seven sections of genus *Arachis*, the Shannon–Weaver diversity index was 0.26. The section *Arachis* exhibited the highest diversity, with a Shannon–Weaver diversity index of 0.46 (Table 2). Subsequently, *Erectoides* and *Procumbentes* exhibited the high diversity with an average Shannon–Weaver diversity index of 0.34. The *Triseminatae* and Synthetic accessions exhibited relatively low diversity, with only 0.48 and 0.49 different alleles, respectively with the 1.09 effective alleles in accessions of both sections and Shannon–Weaver diversity index of 0.08 (Table 2). Within the seven sections, accessions from *Procumbentes* exhibited the highest diversity with a Shannon–Weaver diversity index of 0.18, followed by the accession of *Triseminatae, Erectoides*, and *Heteranthae* that exhibited high diversity with an average Shannon–Weaver diversity index of 0.16, 0.14, and 0.13, respectively. Among the accessions of *Arachis* section, low diversity was very much evident with an average Shannon–Weaver diversity index of 0.09 (Table 3). Shannon–Weaver diversity index was zero in 13 species as these species had only single representation.

**Table 2:** Summary of genetic diversity and effective alleles among different *Arachis* sections.

**Sections**	**Number of accessions**	**No. of different alleles**	**No. of effective alleles**	**Shannon's information index**	**Expected heterozygosity**	**Unbiased expected heterozygosity**
*Arachis*	54	1.95	1.51	0.46	0.30	0.31
*Caulorrhizae*	4	1.05	1.24	0.23	0.15	0.17
*Erectoides*	10	1.56	1.36	0.34	0.22	0.23
*Extranervosae*	3	1.06	1.32	0.28	0.19	0.22
*Heteranthae*	9	1.25	1.28	0.26	0.17	0.18
*Procumbentes*	12	1.67	1.36	0.34	0.22	0.23
*Triseminatae*	2	0.48	1.09	0.08	0.06	0.07
*Synthetics*	2	0.49	1.09	0.08	0.06	0.07
Grand mean	12	1.19	1.28	0.26	0.17	0.19

**Table 3:** Summary of genetic diversity and effective alleles among different *Arachis* species.

**Sections**	**Species**	**Number of accessions**	**No. of different alleles**	**No. of effective alleles**	**Shannon's information index**	**Expected Heterozygosity**	**Unbiased expected heterozygosity**
*Arachis*	*A. batizocoi*	3	0.67	1.11	0.09	0.06	0.07
	*A. benensis*	2	0.59	1.08	0.07	0.05	0.06
	*A. cardenasii*	3	1.12	1.32	0.28	0.19	0.22
	*A. correntina*	1	0.29	1	0	0	0
	*A. decora*	2	0.63	1.13	0.11	0.08	0.1
	*A. diogoi*	2	0.53	1.07	0.08	0.04	0.06
	*A. duranensis*	4	0.99	1.21	0.18	0.12	0.14
	*A. glandulifera*	1	0.48	1	0	0	0
	*A. helodes*	1	0.31	1	0	0	0
	*A. hoehnei*	11	1.42	1.42	0.35	0.24	0.25
	*A. hypogaea*	1	0.51	1	0	0	0
	*A. ipaënsis*	2	0.55	1.07	0.06	0.04	0.05
	*A. kempff-mercadoi*	2	0.83	1.23	0.2	0.14	0.18
	*A. kuhlmannii*	1	0.32	1	0	0	0
	*A. magna*	1	0.3	1	0	0	0
	*A. monticola*	1	0.52	1	0	0	0
	*A. palustris*	5	1.16	1.31	0.27	0.18	0.2
	*A. praecox*	3	0.64	1.15	0.13	0.09	0.11
	*A. simpsonii*	2	0.87	1.22	0.19	0.13	0.18
	*A. stenosperma*	1	0.3	1	0	0	0
	*A. valida*	1	0.41	1	0	0	0
	*A. villosa*	2	0.91	1.28	0.24	0.16	0.22
*Caulorrhizae*	*A. pintoi*	1	0.39	1	0	0	0
*Erectoides*	*A. hermannii*	3	0.89	1.18	0.15	0.1	0.13
	*A. major*	3	0.94	1.22	0.2	0.13	0.16
	*A. oteroi*	1	0.41	1	0	0	0
	*A. paraguariensis*	4	1.05	1.24	0.23	0.15	0.17
	*A. stenophylla*	3	0.88	1.19	0.16	0.11	0.13
*Extranervosae*	*A. lutescens*	2	0.74	1.16	0.14	0.1	0.13
	*A. villosulicarpa*	2	0.49	1.09	0.08	0.06	0.07
*Heteranthae*	*A. dardani*	3	0.68	1.14	0.12	0.08	0.1
	*A. pusilla*	2	0.91	1.22	0.19	0.13	0.17
	*A. sylvestris*	2	0.48	1.09	0.08	0.06	0.07
*Procumbentes*	*A. appressipila*	3	1.19	1.29	0.26	0.18	0.21
	*A. chiquitana*	2	0.67	1.11	0.1	0.07	0.09
	*A. kretschmeri*	2	1.1	1.27	0.23	0.16	0.21
	*A. matiensis*	2	1.07	1.26	0.23	0.16	0.21
	*A. subcoriacea*	3	0.83	1.22	0.2	0.13	0.16
	*A. vallsii*	2	0.63	1.11	0.09	0.06	0.09
*Triseminatae*	*A. triseminata*	2	0.76	1.19	0.16	0.11	0.15

### InDel markers based estimation of genetic relatedness among different *Arachis* species

The analysis of molecular variance (AMOVA) was performed to assess genetic differentiation among sections and species (Table 4). It has been observed that ~15% (*P* = 0.001) molecular variation was attributed to genetic differentiation between the sections while remaining 85% among species within sections. These results indicated the presence of huge genetic variability in genus *Arachis* and between/within sections and species. Of the 86 InDel markers, 32 markers were amplified with the clear cut 50 bp difference between *A. ipaënsis* and *A. duranensis*. Out of these 32 subgenome discriminatory InDels, 16 markers had insertions while remaining 16 had deletions. These markers amplified well in tetraploid and diploid accessions of different sections (Supplementary Table 5). Four InDel markers viz. Ad.A10_44595144 (450 bp), Ai.B06_28217388 (265 bp), Ad.A04_62273881 (200 bp), and Ad.A08_24769399 (260 bp) specifically amplified in only *Arachis* section belonging to A-, B-, and AB- genome (Supplementary Table 6). Similarly, five markers namely Ad.A09_49136207 (275 bp), Ad.A09_49136207 (250 bp), Ai.B10_98116737 (250 bp), Ai.B09_119776002 (250 bp), and Ad.A06_365540 (310 bp) were able to amplify alleles in A, B, and AB genomes of section *Arachis* and P genome of section *Procumbentes*. Interestingly, from the above five markers, the marker Ad.A09_49136207 amplified 850 bp specific allele in some accessions of sections *Caulorrhizae, Extranervosae, Heteranthae*, and *Triseminatae*, which was not detected in remaining accessions of section *Arachis*. Subsequently, three markers namely Ai.B05_59490193 (280 bp), Ad.A01_10274031 (400 bp), and Ad.A10_77068321 (210 bp) could amplify genotypes of sections *Arachis* (A, B, and AB) and *Heteranthae* (H genome), while marker Ad.A10_77068321 (210 bp) could also amplify genotypes of D genome of *Arachis* section (Supplementary Table 6).

**Table 4:** Analysis of molecular variance among section and species.

**Source of variation**	**DF**	**Variance component**	**Variance %**
Among sections	7	4.644	15^**^
Among accessions within species	88	26.446	85^**^
Total	95	31.089	

** *P < 0.001*.

### Alleles specific InDel markers for *Arachis* species and genotypes

For the assessment of genetic diversity among species, the unique alleles provide a good index to discriminate different species in addition to the number of unique alleles in a population which is an elementary estimate of genetic distinctiveness and differentiating degree of speciation of the species/accessions (Chen et al., 2008). Allele bands specific to 41 different species were scored and analyzed. For instance marker, Ai.B04_11232665 amplified a unique 300 bp allele in species *A. batizocoi* of section *Arachis* (B-genome) which did not amplify in any other species (Supplementary Table 7). In addition to this, some markers specifically amplified in some genotypes of different species within and among sections. For instance, marker Ai.B02_41858043 (150 bp) amplified in *A. duranensis* (ICG 8138, A genome), and *A. batizocoi* (ICG 8211, B genome) of *Arachis* section, and *A. stenophylla* (ICG 8215, E genome) of *Erectoides* section. Among these 86 InDel markers, 13 markers amplified multiple alleles across all the 96 accessions. Different amplification events in different species with the same InDel markers manifested the distinct mutational histories of multiple alleles.

### The alleles of cultivated groundnut and its wild relatives

Ascertaining the differences in genetic constitution between *A. hypogaea* and its diploid and tetraploid wild relatives is requisite to empathize the evolution of the cultivated groundnut. In addition to diploid wild progenitor species (*A. duranensis* and *A. ipaënsis)*, the study also included both the tetraploid species i.e., *A. monticola* (AABB) and *A. hypogaea* (AABB) (Supplementary Table 8). In the exploration of the alleles for the cultivated and wild, the alleles Ai.B04_70511351 (200 bp), Ai.B04_11232665 (300), Ai.B03_14363004 (200), Ad.A01_90234020 (450), and Ad.A01_10274031 (250) were found specific to selected wild species *A. stenosperma* (ICG 8137), *A. batizocoi* (ICG 8209 and ICG 13160), *A. duranensis* (ICG 8138), *A. helodes* (ICG 8952) and *A. monticola* (ICG 8135), respectively. Similarly, two common alleles were also reported between wild species viz. Ad.A01_10274031 (400) in *A. kuhlmannii* (ICG 15144) and *A. ipaënsis* (ICG 8206), Ad.A09_119960897 (250) in *A. helodes* (ICG 8952) and *A. palustris* (ICG 15143), Ad.A09_49136207 (750) in *A. helodes* (ICG 8952) and *A. simpsonii* (ICG 15438), and Ai.B02_41858043 (150) in *A. duranensis* (ICG 8138) and *A. batizocoi* (ICG 8211).

### Population structure to unravel genetic architecture

**Figure 3:**
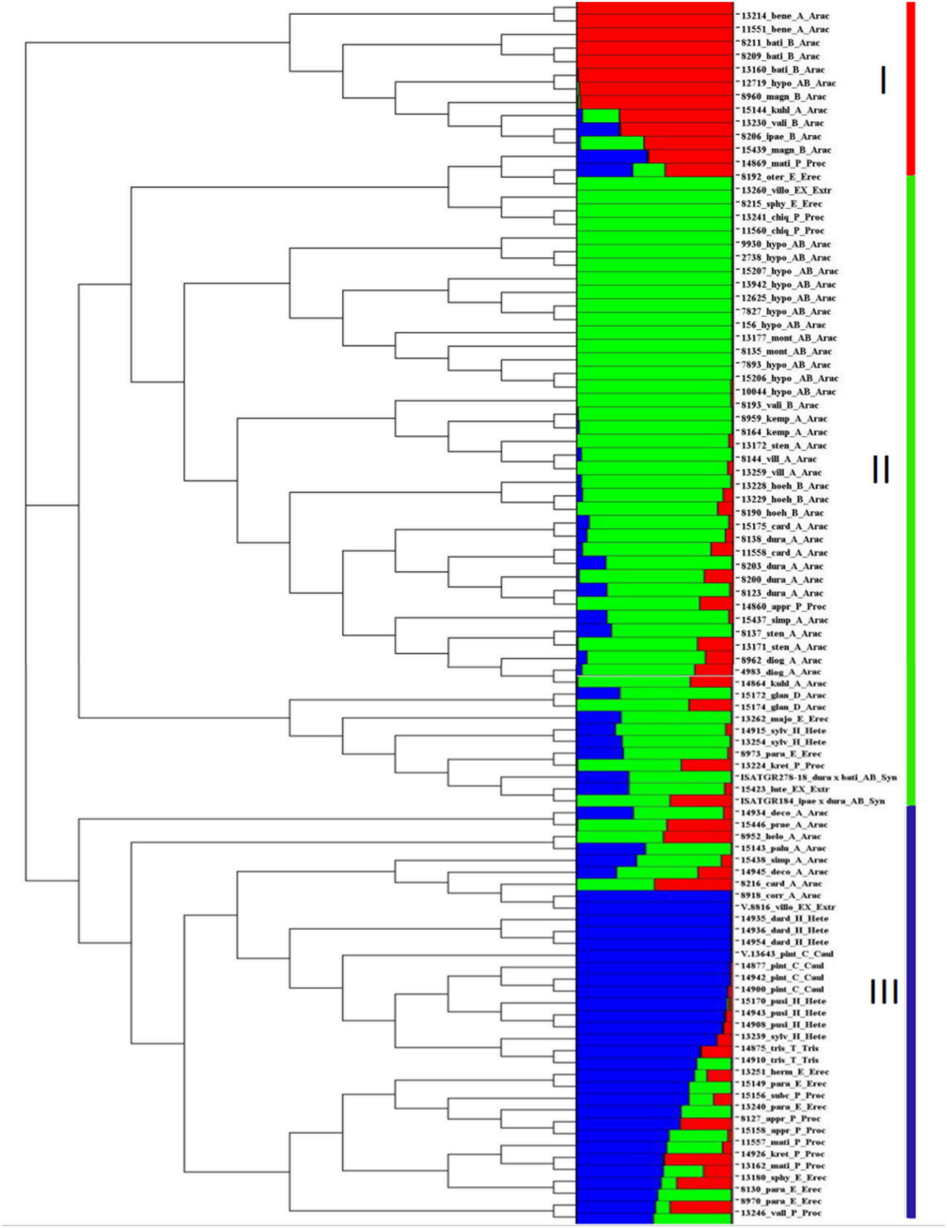
Dendrogram and population structure of 96 cultivated and wild *Arachis* accessions. The 96 *Arachis* accessions were classified into three clusters by structure analysis, I, II, and III, basically accompanying to the phylogenic dendrogram. Red, green, and blue represents the cluster I, II, and III, respectively. The proportion of each color of the horizontal bar represents the assignment possibilities to the specific cluster. The names of accessions and taxonomical information are given next to the horizontal bars, starting with the accession number followed by an abbreviated form of species name followed by respective genomes and sections. *bati, A. batizocoi; bene, A. benensis; card, A. cardenasii; corr, A. correntina; deco, A. decora; chaco, A. chacoense; diog, A. diogoi; dura, A. duranensis; glan, A. glandulifera; helo, A. helodes; hoeh, A. hoehnei; hypo, A. hypogaea; ipaë, A. ipaënsis; kemp, A. kempff-mercadoi; kuhl, A. kuhlmannii; magn, A. magna; mont, A. monticola; palu, A. palustris; prae, A. praecox; simp, A. simpsonii; sten, A. stenosperma; vali, A. valida; vill, A. villosa; pint, A. pintoi; herm, A. hermannii; majo, A. major; oter, A. oteroi; para, A. paraguariensis; sten, A. stenophylla; lute, A. lutescens; villo, A. villosulicarpa; dard, A. dardani; pusi, A. pusilla; sylv, A. sylvestris; appr, A. appressipila; chiq, A. chiquitana; kret, A. kretschmeri; mati, A. matiensis; subc, A. subcoriacea; vall, A. vallsii; trise, A. triseminata; Arac, Arachis; Caul, Caulorrhizae; Erec, Erectoides; Extr, Extranervosae; Hete, Heteranthae; Proc, Procumbentes; Tris, Triseminatae; Synt: Synthetic*.

The population structure of the 96 accessions indicated three clusters i.e., Cluster I (13 accessions), Cluster II (48 accessions) and Cluster III (35 accessions). Cluster I had 13 accessions including 11 accessions from *Arachis* section and 1 accession each from Procumbentes and Erectoides (Figure 3). Cluster II consisted 48 accessions including 35 accessions from the *Arachis* section, 4 accessions from *Procumbentes*, 3 accessions from *Erectoides*, 2 each from *Extranervosae, Heteranthae*, and synthetics (ISATGR278-18 and ISATGR184). Cluster III consisted of 35 accessions which included 8 accessions from the *Arachis* section, 7 accessions from *Procumbentes*, 7 accessions each from *Heteranthae* and *Erectoides*, 4 accessions from *Caulorrhizae*, 2 from *Triseminatae*, and 1 from *Extranervosae* (Figure 3).

### Phylogenetic analyses to establish genetic relatedness

The phylogenetic analysis grouped the 96 *Arachis* accessions into 3 clusters (I, II, and III) corresponding to the structure analysis (Supplementary Table 9; Figure 3). The analysis also showed grouping of the accessions belonging to different genomic sections according to the affinity of their genomes. As expected, all the tetraploid genotypes were grouped together whereas the genotypes belonging to two diploid progenitor genomes (A and B subgenome) grouped separately (Figure 2D). Among other genomes, “P” and “E” genomes clustered together while “C,” “H,” and “T” genomes grouped together showing their genomic similarity with each other. A small separate group was also formed with representative accessions from “Ex,” “P,” and “E” genomes and also synthetics. Also, some accessions from “A” genome grouped with the accessions of “D” and “P” genomes. It was observed that the “D” genome accessions were grouped together with “B” genome indicating higher similarity with each other in comparison to “A” genome. In contrast, surprisingly three B genome accessions belonging to *A. hoehnei* species grouped with cluster dominated by accessions from A genome (Figure 2D).

**Figure 4:**
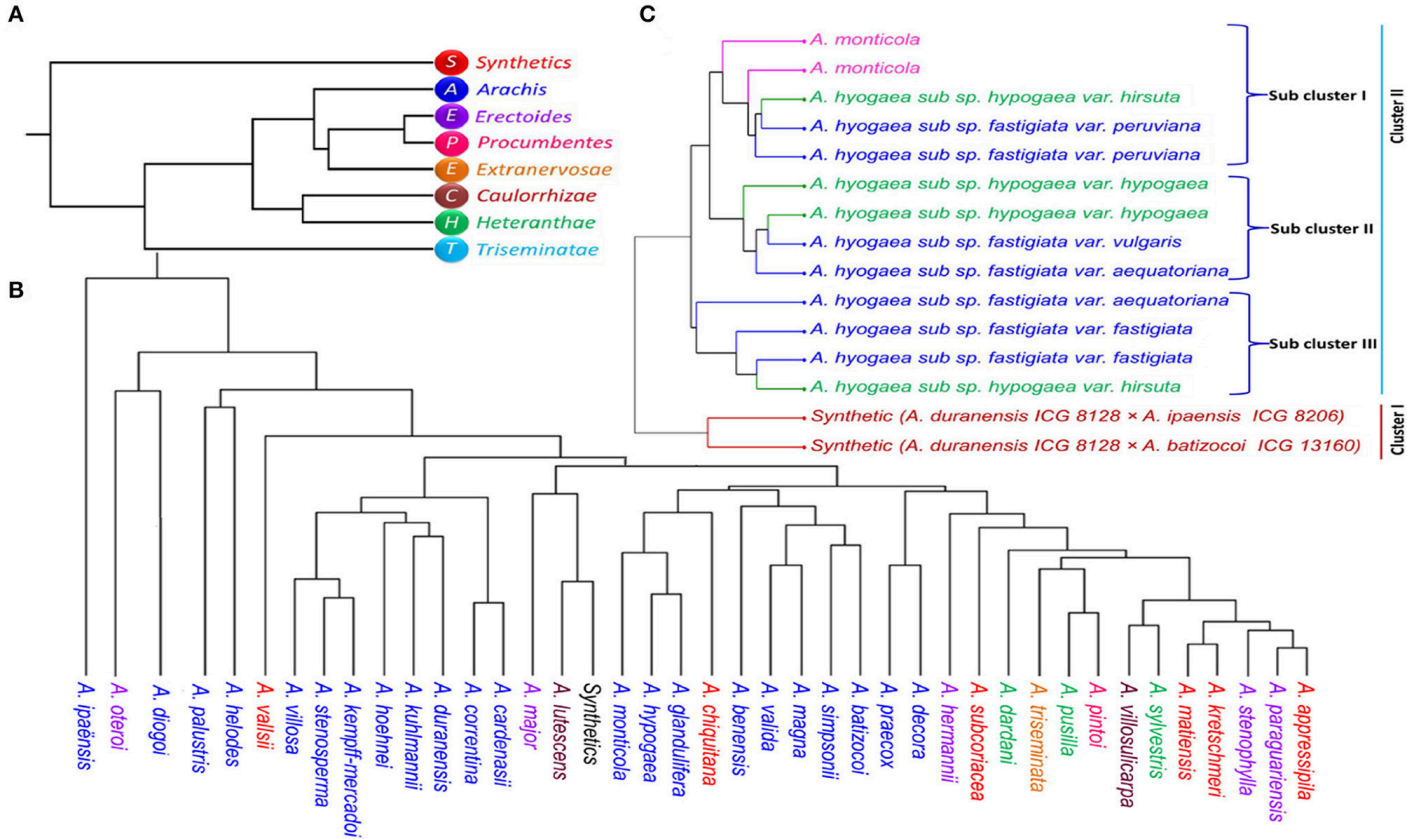
The dendrogram of **(A)** seven sections and synthetics **(B)** 22 species of *Arachis* **(C)** tetraploid genotypes including two synthetics generated from Nei's genetic distance matrix by UPGMA in PHYLIP.

For the analysis of pair-wise relationships between different sections of Arachis, dendrogram based on Nei's distance was constructed. *Erectoides* and *Procumbentes* were clustered together with a genetic distance of 0.011. Synthetics grouped exclusively indicated high distance from other sections (*Triseminatae*: 0.240, *Caulorrhizae*: 0.204, *Heteranthae*: 0.164, *Arachis*: 0.144, *Procumbentes*: 0.117, *Erectoides*: 0.109). Apart from synthetic genotypes, section *Triseminatae* also showed high distance from the other remaining sections with the genetic distances viz. *Arachis*: 0.133, *Caulorrhizae*: 0.127, *Procumbentes*: 0.122, *Extranervosae*: 0.120) (Supplementary Table 10; Figure 4A).

Among the 41 species of *Arachis* genus studied for phylogeny separately, *A. ipaënsis* was highly distant from other species and had greater distance (*A. duranensis*: 0.742, *A. oteroi* 0.704, *A. kempff-mercadoi*: 0.656, *A. major:* 0.605, *A. lutescens*: 0.595, *A. stenosperma*: 0.590, *A. villosa*: 0.548, *A. subcoriacea*: 0.544, *A. palustris*: 0.515). On the other hand, *A. oteroi* also showed greater distance from many species (*A. palustris*: 0.659, *A. hermannii*: 0.574, *A. subcoriacea*: 0.564, *A. batizocoi*: 0.522) (Supplementary Table 11; Figure 4C). *A. paraguariensis* was found closer to *A. appressipila, A. matiensis, A. stenophylla, A. sylvestris*, and *A. kretschmeri* with the shortest distance of 0.039, 0.044, 0.045, 0.051, and 0.053, respectively. Interestingly both the tetraploid species, *A. hypogaea* and *A. monticola* clustered together with mere distance of 0.148 while their diploid progenitors (*A. duranensis*, AA, and *A. ipaënsis*, BB) showed high distance 0.742.

All the tetraploid accessions were evaluated for the differences and relatedness between cultivated, their wild relatives and synthetic genotypes. Two separate groups were formed for the synthetics and cultivated tetraploid genotypes. The second group containing cultivated genotypes clustered according to sub species i.e., sub sp. *fastigiata* and sub sp. *hypogaea*. For greater understanding, hierarchical analysis was performed which identified two major clusters. The first major cluster consisted synthetics and had maximum distance of 0.643 from the second major cluster (Figure 4B, Supplementary Table 12). The second cluster was separated into three sub clusters i.e., sub cluster I consisted *A. monticola*, two *A. hypogaea* sub sp. *fastigiata* var. *peruviana* and strangely one *A. hypogaea* sub sp. *hypogaea* var. *hirsute*. Sub cluster II consisted two *A. hypogaea* sub sp. *fastigiata* var. *fastigiata* with one *A. hypogaea* sub sp. *fastigiata* var. *aequatoriana* and one *A. hypogaea* sub sp. *hypogaea* var. *hirsute*. Sub cluster III consisted of two genotypes from *A. hypogaea* sub sp. *hypogaea* var. *hypogaea* with one *A. hypogaea* sub sp. *fastigiata* var. *vulgaris* and one *A. hypogaea* sub sp. *fastigiata* var. *aequatoriana*.

## Discussion

Over the last decade, next-generation sequencing (NGS) technologies have revolutionized the availability of large-scale genetic markers and their deployment in trait discovery and breeding (Varshney, 2015; Pandey et al., 2016). InDel markers have been deployed in forensic and genetic studies in humans as well as in several plants/crops like wheat, rice, barley, mustard, citrus, tomato, and Arabidopsis (Yang et al., 2014; Lu et al., 2015; Zhou et al., 2015). The availability of draft genome assemblies of groundnut progenitors (Bertioli et al., 2016; Chen et al., 2016) have provided an excellent opportunity for initiating several genomic and genetic studies such as SNP discovery, gene prediction, gene expression, comparative genomics, genetic diversity, genetic mapping, and molecular breeding (Pandey et al., 2016). In this context, this study developed large-scale genome-wide InDel markers and demonstrated their utility in phylogenetic relationship among different species of Arachis genus.

### Large scale genome-wide InDels, an important resource for genetic studies and breeding applications in groundnut

This study discovered large-scale InDels by comparing A subgenome (*A. duranensis*, accession V14167) and B subgenome (*A. ipaënsis*, accession K30076), and detected 5,698 InDels of more than 50 bp sizes indicating an abundance of InDels for genetic and breeding studies in groundnut. Accuracy in InDels identification basically depends on the quality of sequencing data, scheme, and parameters used for data extraction. One and two base pairs InDels were not included between A and B subgenomes in order to avoid over reckoning of small InDels due to sequencing errors as earlier experienced in tomato crop by Yang et al. (2014). In groundnut, identification of InDels have been reported from expressed sequence tags (ESTs) data and was used for studying genetic diversity in cultivated groundnuts (Liu et al., 2015). This is the first report on developing large scale genome-wide InDels using the sequence information of the diploid progenitors of groundnut. Groundnut is usually considered as a less diverse crop, and to explore more studies related to diversity analysis, several marker systems were used from time to time but their numbers were less or not optimum. In this study, we found 40.19% (86 InDels) polymorphism with these InDel markers which were higher than other earlier reported markers viz. start codon targeted polymorphism (SCoT) marker (38.2%) (Xiong et al., 2011); InDels developed from ESTs (33.3%) (Liu et al., 2015); AFLP markers (3.6%) (He and Prakash, 1997); RAPD markers (6.6%) (Subramanian et al., 2000); EST-SSR markers (10.4%) by Liang et al. (2009) and SSR markers (14.5%) Zhao et al. (2012). These InDel markers are a good genomic resource for groundnut research community and can be used in majority of the genetic and breeding applications in groundnut.

### InDel markers provided insights on allele diversity and genetic differentiation

For the better exploitation of wild species genes in groundnut improvement program, knowledge of genetic diversity in the *Arachis* germplasm is essential as indicated in several such studies (Barkley et al., 2007; Angelici et al., 2008). Our findings indicated the presence of lots of diversity within the *Arachis* section and its 23 species. Among the *Arachis* species of section's most diverse accessions was the *A. hoehnei* that belong to B subgenome, which was followed by accessions from the A subgenome (*A. cardenasii, A. palustris*, and *A. villosa*) with the highest Shannon–Weaver diversity index values. The *A. hoehnei* carrying B subgenome while *A. villosa* and *A. cardenasii* (both resistant to rust, LLS, and groundnut rosette) having A genome are considered in secondary gene pool as they are cross-compatible, having chromosome pairing, and hybrid fertility, due to this reason earlier they were considered as probable subgenomes contributors of *A. hypogaea* (Mallikarjuna et al., 2006). Although *A. palustris* is also highly diverse due to aneuploidy species (differences in basic chromosome number) but it would not be cross compatible with *A. hypogaea*, a barrier for the introduction of desirable diverse characters (Lavia, 1998). Differences in the genetic diversity within the *Arachis* section and between species of other sections could be due to the following three reasons or their combination: (1) polyploidization and its events create hurdle in gene mobilization from concerned diploid to cultivated species (Young et al., 1996), (2) combination of self- pollination and polyploidization in immediate past from one or a elite individual(s) of each diploid parental species (Halward et al., 1991), and (3) narrow genetic base induced by consistent use of elite cultivars and less use of exotic germplasm in breeding curricula (Knauft and Gorbet, 1989). This study showed high diversity and researchers need to found better ways for broadening the genetic base by introgressing desired genomic segments from wilds to cultivated groundnut.

### Allele specific indels to different *Arachis* subgenomes supporting existence of K genome

The species specific alleles confer particular species a unique identity in the population. Out of 86 InDel markers examined, 9 markers amplified 9 alleles, which were specific to genotype/accession and dissimilar for *Arachis* sections. The unique alleles were observed in the species/accessions indicating the various degrees of evolution and diversity of these species/accessions (Chen et al., 2008). We identified few markers that were specific to a particular genome like four markers amplified only *Arachis* section (A-, B-, AB- genome) which were not amplified in any other sections (representative of other than A-, B-, AB- genome). This indicated occurrence of new recombination events that might have shuffled the genomic sequences and created insertions and deletions sites due to domestication. Likewise, some markers amplified in *Caulorrhizae, Extranervosae, Heteranthae*, and *Triseminatae* sections, but not amplified in any accessions from *Arachis* section, these wild crop relatives of *Arachis* are endemic to South America, occurring in Bolivia, Argentina, Brazil, Paraguay, and Uruguay and are a rich source of specific alleles (Valls et al., 1985). This suggested that some genes/alleles specific to them were lost after natural selection or domestication events. In contrast to the markers amplified in *Arachis* section and as well as in section *Procumbentes and Heteranthae*. This result revealed sharing of some large genomic sequences between *Arachis* and *Procumbentes* sections, *Arachis* and *Heteranthae* sections which was not fully recombined during domestication and remained conserved. Likewise, two genotypes of *A. batizocoi* had a specific allele that was not found in any other genotypes or species. Our study is in quite an accordance with the earlier study (Leal-Bertioli et al., 2014) which claimed this species might have another genome “K” with more similarity to “B” genome. For getting insights into genetic constitution among *A. hypogaea* and its diploid and tetraploid wild congenators, we also considered specific alleles between them. We found five genotypes belonging to wild diploid species with one exception i.e., *A. monticola* with specific alleles within *Arachis* section. This indicated that these alleles were highly conserved in wild relatives and emerged during the speciation and evolution of groundnut but were restricted to wild or lost in domestication events. These wild resources can be a good source of mobilizing specific alleles from wild to diversify the genetic base of cultivated gene pools and to enrich economically significant traits.

### InDels established genomic affinities among diverse germplasm

The InDel-based phylogenetic study grouped all the seven taxonomic sections based on their genomic affinities with the exception of synthetic genotypes which grouped distinctly from all the sections. *Arachis* species is considered to be the most diverse, holding both annual and perennial species and distinct chromosome numbers, karyotype structures and ploidy levels as it was a group apart but very close to *Erectoides* and *Procumbentes* Krapovickas and Gregory, 1994). On the other hand, *Heteranthae* and *Caulorrhizae* grouped together indicating that some species of the sections *Heteranthae* and *Caulorrhizae* may be capable of producing hybrids with *Arachis* section while a substantial genetic isolation persists with the other sections (Bertioli et al., 2016). According to Krapovickas and Gregory (1994) sections, *Extranervosae* and *Triseminatae* are the most detached sections, however, their evolutionary place is yet to be decided (see Stalker, 2017). This study confirms the above assumption for *Triseminatae* as the most isolated section from the remaining sections. On the other hand, this study contradicts in case of *Extranervosae* which was found close to the *Arachis, Procumbentes*, and *Erectoides*. Nevertheless, the recent phylogenetic studies based on ribosomal DNA (rDNA) suggested sections *Heteranthae, Extranervosae*, and *Triseminatae* to be most primitive and section *Arachis* to be most recent while sections *Procumbentes, Caulorrhizae, Erectoides, Rhizomatosae*, and *Trierectoides* found in between these sections (Bechara et al., 2010; Wang et al., 2010). This study with the InDel markers indicates sections *Heteranthae* and *Triseminatae* to be the most primitive including *Caulorrhizae;* sections *Erectoides* and *Procumbentes* as intermediate and section *Arachis* as the most recent in origin.

The topology of the UPGMA dendrogram for all 44 species used in this study generated from InDel markers revealed that during the whole evolutionary courses of *Arachis* to date, there have been new recombinants occurring due to regular and frequent deletion process. Due to above possible reasons, all the species show higher affinity to A genome rather than B genome representative *A. ipaënsis*. This also indicated that the other B genome species, grouped close to A genome representative *A. duarnensis*, are distinct from *A. ipaënsis* due to greater difference in accumulation of deletions in the genome. The analysis of InDel markers showed grouping of species from D, K, and F genomes together with A genome species. The position of *A. benensis* distant from the B genome biological group also gives support to the validity of the F genome assignment. The position of the D, K, and F genomes closer to the A than to the B genome is worthy of further investigation.

### *A. monticola*: a true wild species escaped away from cultivation or ancestor?

Looking insights into the relationship of tetraploid genotypes/accessions based on the InDel markers, both the synthetic genotypes grouped separately as they were newly created and indicated their diverse genetic makeup. In addition, the dendrogram clearly showed that *A. monticola* and cultivated *A. hypogaea* (all botanical type) were grouped together with less or null genetic distance due to their close affinity. The complete homology observed here in the insertion and deletion sequences that manifested *A. monticola* and *A. hypogaea* tetraploid taxa very near and similar genome composition. The current finding also justifies the high rate of crossability and achieving fertile progenies reported by Krapovickas and Gregory (1994). Albeit, the dendrogram of tetraploid species, showed that *A. monticola* grouped separately from the other botanical types, which affirms the belief that it is a separate species from *A. hypogaea*. In a study, *A. monticola* was believed as a true wild species that had got away from cultivation which was not considered as a form of *A. hypogaea* (Krapovickas and Gregory, 1994; Bechara et al., 2010). This finding is also supported by the studies done on the basis of fruit structure which narrowly separate each seed, *A. monticola* was considered a discrete species from *A. hypogaea* (Krapovickas and Gregory, 1994). This attribute was not found in any cultivated groundnut and is conceived as a naive feature in the genus. These observations affirm the possibility that *A. monticola* is the immediate wild ancestor or an introgressive derivative between the *A. hypogaea* and wild species as reported in earlier studies (Gregory and Gregory, 1976; Moretzsohn et al., 2004; Koppolu et al., 2010).

## Conclusion

The InDels are the second most abundant structural variations across the genome after SNPs and can serve as genetic markers for conducting genetic studies in labs with small to medium scale genotyping facility, especially with higher length polymorphism. This study successfully identified 515,223 InDels distributed across groundnut genome and designed primers for 5,698 InDels with >50 bp size. Further, randomly selected InDel markers were validated for their functionality and usefulness in studying the genetic relationship in a very diverse germplasm sets. The information on InDel markers is a very useful genomic resource for the groundnut research community for using them in an array of genetic and breeding applications.

## Author contributions

MKV and MKP: performed most of the experiments; SMK: performed InDel marker discovery and primer designing; MS, TN, and YS: generated genotyping data on diverse germplasm panel; MKV, MS, and VG: analyzed genotyping data and conducted genetic analysis; MKV and MKP: interpreted the results; MKV: drafted the MS; MKP and RKV: improved the manuscript; MKP and RKV: conceived, designed, and supervised the study and finalized the manuscript.

### Conflict of interest statement

The authors declare that the research was conducted in the absence of any commercial or financial relationships that could be construed as a potential conflict of interest.
