# Supplementary material for: Genome-Wide Discovery and Deployment of Insertions and Deletions Markers Provided Greater Insights on Species, Genomes, and Sections Relationships in the Genus Arachis
Source: Front Plant Sci. 2017 Dec 19;8:2064. doi: 10.3389/fpls.2017.02064 (PMC5742254; doi:10.3389/fpls.2017.02064)
Supplement: Supplementary file 2 [file Table2.DOCX]

**Supplementary Table 2. In house Perl script used for genome-wide InDel discovery**

Script Description:

pro_maf_align.pl

For extracting data ended with maf from MUGSY, run “perl pro_maf_align.pl mugsy.maf output1”

extr_flanking_indel.pl

for extracting flanking sequences, run “perl extr_flanking_indel.pl file1 file2 output1”

extr_blast_best.pl

for extracting blast output. run “perl extr_blast_best.pl blast.out output2”

select_deletion_d.pl OR select_insertion_d.pl

for extracting the best InDel. run “perl select_insertion_d.pl output2 output3”

extr_seq_for_primer.pl

for extracting flanking seqeunces for primer design. run “perl extr_seq_for_primer.pl output3 output”

primer3_20111017.pl

for primer design. run “perl primer3_20111017.pl indel.flanking.fa indel_primer.txt”

extr_primer3.pl

for extracting primer sequences. run “perl extr_primer3.pl -p reult.txt”

================================================================================================

Script 1: pro_maf_align.pl

================================================================================================

#!/usr/bin/perl -w

use strict;

open(INPUT,$ARGV[0])||die "Cant open the maf alignment file.\n";

open(OUT,">".$ARGV[1]);

$/="##maf version=1 scoring=single_cov2";

my $t=<INPUT>;

print $t,"\n";

my $info=<INPUT>;

print "start\n";

my $identical=0;

my $d_gap=0;my $w_gap=0;my $snp=0;

my $insert=0;my $deletion=0;

my $insert_len=0;my $deletion_len=0;

#open(TTT,">ttttt");

my $ggg=0;

while($info=~/a\s+(score=\d+.+)\ns[\t\s]+(chrp\S+)[\t\s]+(\d+)[\t\s]+(\d+)[\t\s]+([+-])[\t\s]+(\d+)[\t\s]+(\S+)\ns\s+(pimp\S+)\s+(\d+)\s+(\d+)\s+([+-])\s+(\d+)\s+(\S+)\n\n/g)

{

#print TTT $&,"\n";

$ggg++;

my $score=$1;my $d_t=$2;my $d_start=$3;my $d_aln_len=$4;my $d_length=$6;my $w_t=$8;my $d_align=$7;my $w_aln_len=$10;my $w_length=$12;my $w_align=$13;my $strand=$11;

# my $w_start=$9;

# print "iner\n";

my $w_start=0;

if($11 eq '-')

{

$w_start=$12-$9+1;

}

else
